# Molecular Dynamics Simulations of the Permeation of Bisphenol A and Pore Formation in a Lipid Membrane

**DOI:** 10.1038/srep33399

**Published:** 2016-09-15

**Authors:** Liang Chen, Junlang Chen, Guoquan Zhou, Yu Wang, Can Xu, Xiaogang Wang

**Affiliations:** 1 School of Sciences, Zhejiang A & F University, Lin’an 311300, China; 2 School of Environmental and Chemical Engineering, Shanghai University, 99 Shangda Road, Shanghai 200444, China; 3 Key Lab for Magnetism and Magnetic Materials of MOE, Lanzhou University, Lanzhou 73000, China

## Abstract

Bisphenol A (BPA) is particularly considered as one of the most suspicious endocrine disruptors. Exposure to BPA may bring about possible human toxicities, such as cancerous tumors, birth defects and neoteny. One of the key issues to understand its toxicities is how BPA enters cells. In this paper, we perform molecular dynamics simulations to explore the interactions between BPA and a phospholipid membrane (dipalmitoylphosphatidylcholine, DPPC bilayer). The simulation results show that BPA can easily enter the membrane from the aqueous phase. With the increasing concentrations of BPA in the membrane, BPA tends to aggregate and form into cluster. Meanwhile, several DPPC lipids are pulled out from each leaflet and adsorbed on the cluster surface, leading to pore formation. Detailed observations indicate that the lipid extraction results mainly from the dispersion interactions between BPA cluster and lipid tails, as well as weak electrostatic attractions between lipid headgroups and the two hydroxyl groups on BPA. The lipid extraction and pore formation may cause cell membrane damage and are of great importance to uncover BPA’s cytotoxicity.

Bisphenol A (BPA), 2, 2-bis (4-hydroxyphenyl) propane, an important chemical material, is widely used to produce epoxy resins and polycarbonates^1^,^2^. It is also highlighted as one endocrine disrupting chemical^3^,^4^,^5^, which may cause the function abnormalities of human endocrine system^6^,^7^,^8^, including mimicking estrogen^9^,^10^, occupying the hormone receptors, and disrupting hormone synthesis, secretion^11^.

Prior to affect the endocrine system, BPA should pass through the cell membrane. Therefore, it is fundamentally essential to understand the translocation of BPA across a cell membrane and its effects therein. However, the current studies mainly focus on the toxicity of BPA and how to detect^12^,^13^,^14^,^15^, and eliminate BPA from water solution^16^,^17^,^18^,^19^. Few attention is paid to the interactions between BPA and cell membrane^20^,^21^.

Here, we use molecular dynamics simulation to investigate the penetration of BPA and its cluster from water into the lipid bilayer and the aggregation of BPA in the bilayer center. The results show that BPA can readily enter the membrane and is trapped in the bilayer close to the lipid headgroups. Under the high concentration of BPA in the water, BPA will form into cluster before entering the membrane. When aggregated in the membrane, BPA cluster can extract lipids and adsorb them on its surface, resulting in pore formation. Such effect has not been reported previously, and it may offer new insights for the toxicities of BPA.

## Computational Methods

Molecular dynamics (MD) simulations were performed on the hydrated DPPC bilayer with BPAs. The fully hydrated bilayer, composed of 128 DPPC lipids and 5800 water molecules, was developed by Tieleman and Berendsen^22^. The force field parameters for DPPC lipids were taken from Berger *et al.*^23^. The geometry of BPA was optimized by density functional theory (DFT) method at the B3LYP/6-31G (d, p) level^24^. The topology of BPA was created by PRODRG server^25^. However, the partial charges assigned by PRODRG were found to result in wrong water solubility and unrealistic partitioning between water and membrane. We therefore resorted to Mulliken partial charges obtained after DFT calculations, which were compatible with Berger lipid force field.

All MD simulations were carried out under the isothermal-isobaric (NPT) ensemble using the Gromacs package 4.5.5^26^,^27^ and GROMOS53a6 force field^28^. Periodic boundary conditions were employed in all directions. The vdW interactions were treated with smooth cutoff at a distance of 12 Å, whereas the particle-mesh Ewald method was adopted to calculate the long-range electrostatic interactions^29^,^30^. Water was represented by the SPC model^31^. The temperature was kept stable at 323 K using the V-rescale thermostat^32^ and the pressure was controlled semi-isotropically by a Berendsen barostat^33^. Bond lengths within BPA/DPPC and water molecules were constrained by the LINCS and the SETTLE algorithms^34^,^35^, respectively.

The free energy of BPA across the bilayer was computed from the potential of mean force (PMF) using umbrella sampling^36^. First, we conducted steered MD simulation to pull the molecule from the aqueous phase to the middle of the membrane. Then, 30 configurations were generated along the z-axis direction (reaction coordinate). The z coordinates of COM distance between BPA and membrane in each configuration differed by about 0.1 nm. Each window was equilibrated for 5 ns and a production run of 5 ns was continued for sampling. Eventually, the PMF profile was obtained by the Weighted Histogram Analysis Method (WHAM)^37^, implemented in the GROMACS package as ‘g_wham’^38^. The convergence and sufficiency and of each sampling were presented in the Supplementary Information Figures S1 and S3.

## Results and Discussion

### The permeation of BPA in the membrane

**Figure 1:**
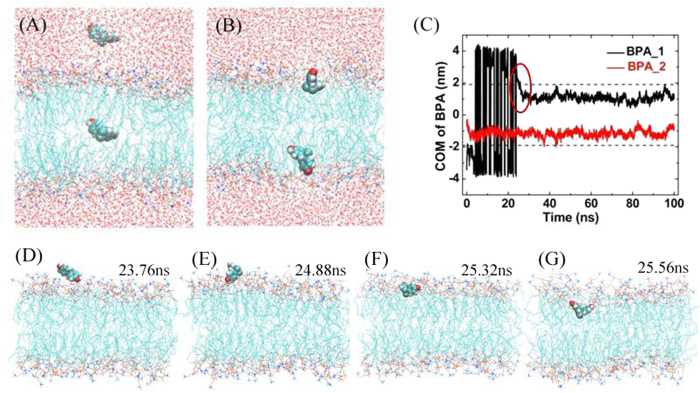
A trajectory of the two BPA molecules in the membrane. (**A**) The initial configuration. (**B**) The final snapshot. (**C**) The z-coordinates of the COM of BPA vs. simulation time, where the center of the bilayer is fixed at z = 0 nm, and the two dashed lines represent the interfaces between water and lipid headgroups. (**D–G**) Snapshots specify the dynamic process of BPA entering the bilayer, where water is not shown for clarity.

Because of the extremely low water solubility, we only put one BPA in the aqueous phase and one in the bilayer center (see Fig. 1A) to look for the preferable location of the molecules. Figure 1 showed the initial and final structures and the time evolution of the z-coordinates of their COMs. During the first 25 ns, the outer BPA just moved randomly in the water. However, at *t*~25.32 ns, the outer BPA rapidly entered the bilayer and then stayed at the interface between water and the lipid headgroups. The detailed dynamic process of the outer BPA entering the lipid bilayer was presented in Fig. 1D–G. First, BPA was adsorbed on the surface of bilayer because of the electrostatic interactions between the two hydroxyl groups of BPA and the lipid headgroups (see Fig. 1D, *t* = 23.76 ns). Due to the hydrophobic interactions between BPA and lipid tails, the main body of BPA rotated and then was pulled into the lipid tails (see Fig. 1E–G), while the hydroxyl groups were still near the headgroups. Such location and orientation had no essential changes but slight fluctuations for the rest of the simulation. Meanwhile, the inner BPA went straight and quickly to another interface and remained there with the similar location and orientation till the end of simulation. The two symmetric locations were in line with NMR determination^39^. We have rerun the system with more accurate partial charges, obtained by DFT calculation at wb97xd/6 − 31 + g(d, p) level. As shown in Figure S4 in the Supplementary Information, the results are in good agreements with those in Figure 1.

**Figure 2:**
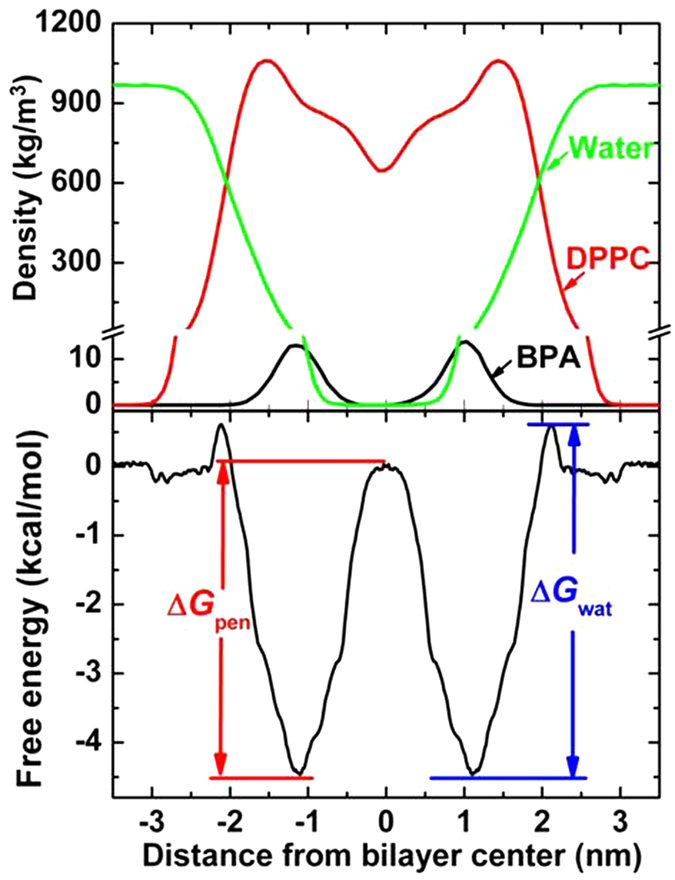
(**A**) Mass density profiles of BPA (black), DPPC (red) and water (green). (**B**) Potential of mean force (PMF) of BPA across the membrane. The labeled ΔG_wat_ and ΔG_pen_ denote water/lipid barrier and the bilayer center penetration barrier.

The translocation of BPA can be more quantitatively illustrated by the PMF of BPA at different transmembrane positions, as shown in Fig. 2B. The PMF of BPA in the aqueous phase far away from the interface was defined as zero. We observed that BPA penetrating into the bilayer should overcome an energy barrier, though it was not high (~0.6 kcal/mol). However, the free energy difference between BPA at the center and at the interface reached 5.1 kcal/mol (ΔG_pen_), resulting in its fast moving towards the lipid headgroups. The minimum of the PMF was −4.5 kcal/mol at the symmetric positions ± 1.1 nm from the bilayer center. Mass density profile (see Fig. 2A) showed clearly that the preferable locations of BPA were symmetric and close to the lipid headgroups (the densest region of the membrane). These two locations were in good agreement with the two minima of the PMF, implying that they were energetically favorable.

**Figure 3:**
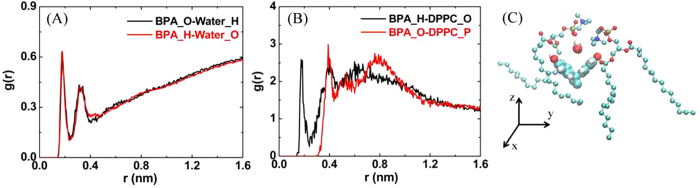
The detailed interactions among DPPC, BPA and water. (**A**) RDF between BPA and lipid headgroups. (**B**) RDF between BPA and water. (**C**) A configuration showing the orientation of BPA, its two nearest and a water molecule bridged between BPA and lipid headgroups.

Figure 3 showed the detailed interactions among DPPC, BPA and water. The orientation of BPA in the membrane was that its hydroxyl groups pointed to the hydrophilic interface and the rest of the molecule was immersed in the lipid tails (see Fig. 3C), due to the following two facts: 1) the electrostatic interactions between the two hydroxyl groups on BPA and the lipid headgroups; 2) the hydrophobic interactions between the two benzene rings and methyl groups on BPA and the lipid tails. Radial distribution functions (RDF) were calculated to elucidate the former. The first peak between polar hydrogen atoms on BPA and oxygen atoms on phosphate groups (Fig. 3A, black line, at ~0.18 nm) corresponded to a hydrogen bond, while the first peak between oxygen atoms on BPA and phosphorus atoms on phosphate groups (Fig. 3A, red line) was at ~0.39 nm, close to their collision radii. These peaks indicated that the hydroxyl groups on BPA strongly interacted with the polar lipid headgroups.

Water molecules could easily penetrate into the interspaces between lipid headgroups, and act as bridges between lipid headgroups as well as between BPA and headgroups. Figure 3B showed the RDF of water molecules around BPA. The first (at ~0.18 nm) and second peak (at ~0.35 nm) represented the hydrogen bonds, meaning that water in the membrane preferred to surround BPA. After this, the RDF rose gradually with the increasing distance, since more and more water molecules were included out of the membrane.

### The permeation of BPA cluster in the membrane

**Figure 4:**
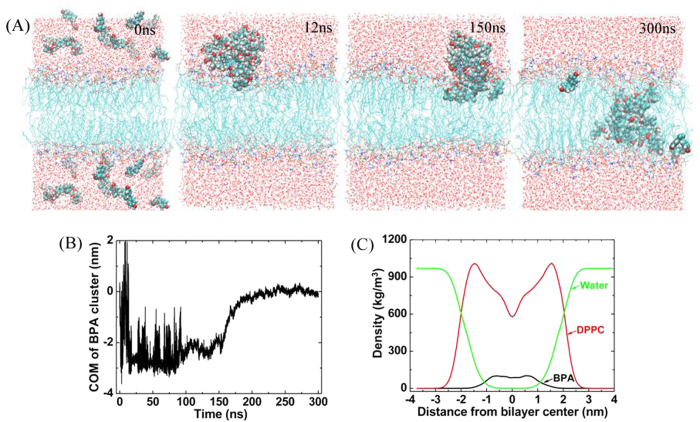
Trajectory of BPA cluster entering the membrane. (**A**) Snapshots taken at 0 ns, 12 ns, 150 ns and 300 ns, respectively. (**B**) Time evolution of the COM of BPA cluster. (**C**) Mass density profile of the system in the last 50 ns.

We then increased the concentration of BPA in the water to 0.43 mol%, in which mol% was defined as the number of BPA divided by the sum of BPA and water molecules. As shown in Fig. 4A, BPA formed into cluster quickly at *t* = 12 ns, and then moved randomly in the water. At about *t* = 150 ns, BPA cluster began to slowly enter the membrane. Figure 4B showed that the difference between the COMs of BPA cluster and lipid bilayer was close to zero after entering the membrane, since the COM of BPA cluster was near the bilayer center (see Fig. 4A, snapshot at *t* = 300 ns). The BPA cluster was kept almost intact except that 3 BPA molecules went away from the cluster. Mass density profile (Fig. 4C) also showed that different from single BPA in the membrane, BPA cluster preferred to stay in the middle of the bilayer. That is to say, BPA presents low lipid solubility.

**Figure 5:**
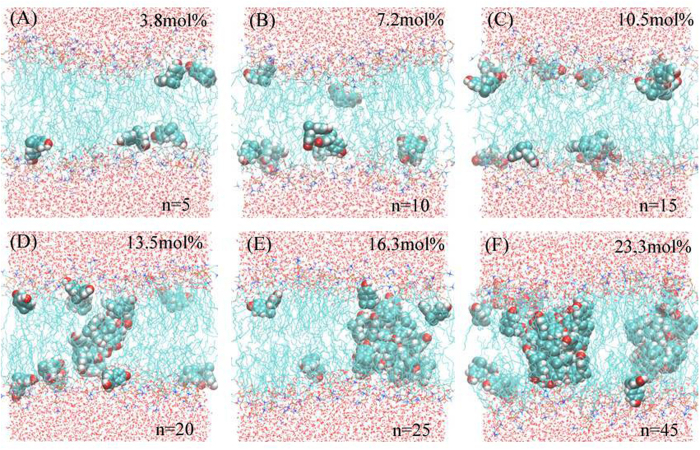
Low lipid solubility of BPA in the membrane, in which the number of BPA molecules was set according to the six fixed concentrations, namely (**A**) 3.8 mol%, (**B**) 7.2 mol%, (**C**) 10.5%, (**D**) 13.5%, (**E**) 16.3% and (**F**) 23.3 mol%.

To confirm the low solubility of BPA in the lipid membrane, we randomly embedded BPA molecules in the bilayer center with different concentrations, namely 3.8 mol%, 7.2 mol%, 10.5 mol%, 13.5 mol%, 16.3 mol% and 23.3 mol%, Correspondingly, the number (abbreviated to *‘n’*) of BPA was *n* = 5, 10, 15, 20, 25 and 45. Here, mol% was defined as the number of BPA divided by the sum of BPA and DPPC molecules. The results were given in Fig. 5. At extremely low concentration, BPA could disperse in the membrane (see Fig. 5A) and BPA was close to the lipid headgroups. Then, BPA began to aggregate into small clusters, when the concentration was about 7.2~13.5 mol% (see Fig. 5B–D). And BPA formed into big clusters, when the concentration was more than 16.3 mol% (Fig. 5E,F). In general, BPA has weak lipid solubility according to our simulation results.

**Figure 6:**
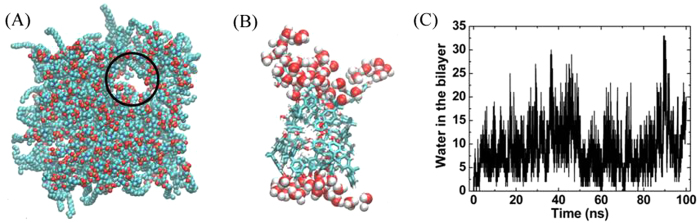
Pore formation in the DPPC bilayer. (**A**) Top view of the bilayer, where black circle specifies the water pore. (**B**) The structure of BPA cluster and water molecules surrounded it. (**C**) The number of water molecules in the membrane.

Interestingly, it was found that pore formed at the entrance (see Fig. 6A) where BPA cluster entered the membrane. And water molecules flew in and filled the hole. Detailed observation showed that these holes were located on both sides of BPA cluster, and several lipids broke away from the bilayer and were adsorbed on the surface of the cluster. The pore formation was also found in the systems, in which the concentration of BPA in the membrane was more than 16.3 mol%. It has been reported that the dispersion interactions between *sp*^2^ carbon atoms and lipid tails are stronger than the self-attraction among the lipids in the bilayer^40^. Clearly, this strong attraction between BPA cluster and lipids originates from such dispersion interactions between the benzene rings on BPA and lipid tails. Figure 6C showed the number of water molecules in the bilayer as a function of simulation time. These water molecules were mainly distributed on both sides of the BPA clusters (see Fig. 6B), where the holes existed.

## Conclusions

The translocation and aggregation of BPA in a DPPC bilayer have been investigated by molecular dynamics simulations. Under low concentration, BPA can readily penetrate into the membrane and prefer to stay at the interface between water and lipids, with its hydroxyl groups towards the lipid headgroups and its carbon rings in the lipid tails. This location and orientation enable electrostatic interactions between the hydroxyl groups of BPA and the polar headgroups of DPPC as well as water, which can be confirmed by hydrogen bonds found in the RDFs. With high concentration, BPA clusters first and then enters the membrane, leaving a hole at the entrance. The aggregation of BPA in the membrane can also cause pore formation, since several lipids are extracted from each leaflet and adsorbed on the cluster surface. The dispersion interactions between lipid tails and the benzene rings on BPA as well as electrostatic attractions among their polar groups contribute to the lipid extraction. The results provide detailed atomic mechanism of the interactions between BPA and a model cell membrane. And BPA may possess potential cytotoxicity when clustered in the membrane.

## Additional Information

**How to cite this article**: Chen, L. *et al.* Molecular Dynamics Simulations of the Permeation of Bisphenol A and Pore Formation in a Lipid Membrane. *Sci. Rep.* **6**, 33399; doi: 10.1038/srep33399 (2016).

## Supplementary Material

Supplementary Information

## References

[b1] Ahmad S., Gupta A. P., Sharmin E., Alam M. & Pandey S. K. Synthesis, characterization and development of high performance siloxane-modified epoxy paints. Prog. Org. Coat. 54, 248–255 (2005).

[b2] And C. F. A. & Kremer K. Combined coarse-grained and atomistic simulation of liquid bisphenol A-polycarbonate: liquid packing and intramolecular structure. Macromolecules 36, 260–267 (2003).

[b3] Rubin B. S. Bisphenol A: an endocrine disruptor with widespread exposure and multiple effects. J. Steroid. Biochem. Mol. Biol. 127, 27–34 (2011). 21605673 10.1016/j.jsbmb.2011.05.002

[b4] Vandenberg L. N., Maffini M. V., Sonnenschein C., Rubin B. S. & Soto A. M. Bisphenol-A and the great divide: a review of controversies in the field of endocrine disruption. Endocr. Rev. 30, 75–95 (2009). 19074586 10.1210/er.2008-0021 PMC2647705

[b5] Tsai W. T. Human health risk on environmental exposure to Bisphenol-A: a review. J. Environ. Sci. Heal. C 24, 225–255 (2006). 10.1080/10590500600936482 17114111

[b6] Kang J. H., Kondo F. & Katayama Y. Human exposure to bisphenol A. Toxicology 226, 79–89 (2006). 16860916 10.1016/j.tox.2006.06.009

[b7] Staples C. A., Dorn P. B., Klecka G. M., O’Blook S. T. & Harris L. R. A review of the environmental fate, effects, and exposure of bisphenol A. Chemosphere 36, 2149–2173 (1998). 9566294 10.1016/s0045-6535(97)10133-3

[b8] Dekant W. & Volkel W. Human exposure to bisphenol A by biomonitoring: methods, results and assessment of environmental exposures. Toxicol. Appl. Pharmacol. 228, 114–134 (2008). 18207480 10.1016/j.taap.2007.12.008

[b9] Welshons W. V. *et al.* Large effects from small exposures. I. mechanisms for endocrine-disrupting chemicals with estrogenic activity. Environ. Health Persp. 111, 994–1006 (2003). 10.1289/ehp.5494 PMC1241550 12826473

[b10] Alonso-Magdalena P. *et al.* Bisphenol-A acts as a potent estrogen via non-classical estrogen triggered pathways. Mol. Cell. Endocrinol. 355, 201–207 (2012). 22227557 10.1016/j.mce.2011.12.012

[b11] Wolstenholme J. T., Rissman E. F. & Connelly J. J. The role of Bisphenol A in shaping the brain, epigenome and behavior. Horm. Behav. 59, 296–305 (2011). 21029734 10.1016/j.yhbeh.2010.10.001 PMC3725332

[b12] Deng P., Xu Z. & Kuang Y. Electrochemically reduced graphene oxide modified acetylene black paste electrode for the sensitive determination of bisphenol A. J. Electroanal. Chem. 707, 7–14 (2013).

[b13] Zhang Y. *et al.* Electrochemical sensor for bisphenol A based on magnetic nanoparticles decorated reduced graphene oxide. Talanta 107, 211–218 (2013). 23598214 10.1016/j.talanta.2013.01.012

[b14] Zhu Y. *et al.* Building an aptamer/graphene oxide FRET biosensor for one-step detection of bisphenol A. ACS Appl. Mater. Inter. 7, 7492–7496 (2015). 10.1021/acsami.5b00199 25799081

[b15] Dai H. *et al.* Amplified electrochemiluminescence of lucigenin triggered by electrochemically reduced graphene oxide and its sensitive detection of bisphenol A. Anal. Methods 6, 4746–4753 (2014).

[b16] Xu J., Wang L. & Zhu Y. Decontamination of bisphenol A from aqueous solution by graphene adsorption. Langmuir 28, 8418–8425 (2012). 22571829 10.1021/la301476p

[b17] Zhang Y. *et al.* Recyclable removal of bisphenol A from aqueous solution by reduced graphene oxide-magnetic nanoparticles: adsorption and desorption. J. Colloid Interf. Sci. 421, 85–92 (2014). 10.1016/j.jcis.2014.01.022 24594036

[b18] Xu J. & Zhu Y. F. Elimination of bisphenol A from water via graphene oxide adsorption. Acta Phys. -Chim. Sin. 29, 829–836 (2013).

[b19] Chang H. S., Choo K. H., Lee B. & Choi S. J. The methods of identification, analysis, and removal of endocrine disrupting compounds (EDCs) in water. J. Hazard Mater. 172, 1–12 (2009). 19632774 10.1016/j.jhazmat.2009.06.135

[b20] Takechi Y., Shintani Y. & Kimoto D. Regulation of phospholipid protrusion in the cell sized vesicle by hydrophobic bisphenol A. Membrane 40, 38–45 (2015).

[b21] Altamirano G. A. *et al.* Milk lipid composition is modified by perinatal exposure to bisphenol A. Mol. Cell. Endocrinol. 411, 258–267 (2015). 25976663 10.1016/j.mce.2015.05.007

[b22] Tieleman D. P. & Berendsen H. J. C. Molecular dynamics simulations of a fully hydrated dipalmitoylphosphatidylcholine bilayer with different macroscopic boundary conditions and parameters. J. Chem. Phys. 105, 4871–4880 (1996).

[b23] Berger O., Edholm O. & Jahnig F. Molecular dynamics simulations of a fluid bilayer of dipalmitoylphosphatidylcholine at full hydration, constant pressure, and constant temperature. Biophys. J. 72, 2002–2013 (1997). 9129804 10.1016/S0006-3495(97)78845-3 PMC1184396

[b24] Becke A. D. Density-functional exchange-energy approximation with correct asymptotic behavior. Phys. Rev. A 38, 3098–3100 (1988). 10.1103/physreva.38.3098 9900728

[b25] Schuttelkopf A. W. & van Aalten D. M. F. PRODRG: a tool for high-throughput crystallography of protein-ligand complexes. Acta Cryst. D 60, 1355–1363 (2004). 15272157 10.1107/S0907444904011679

[b26] Berendsen H. J. C., Spoel D. v. d. & Drunen, R. v. GROMACS: A message-passing parallel molecular dynamics implementation. Comput. Phys. Commun. 91, 43–56 (1995).

[b27] Hess B., Kutzner C., van der Spoel D. & Lindahl E. GROMACS 4: Algorithms for Highly Efficient, Load-Balanced, and Scalable Molecular Simulation. J. Chem. Theory Comput. 4, 435–447 (2008). 26620784 10.1021/ct700301q

[b28] Oostenbrink C., Villa A., Mark A. E. & van Gunsteren W. F. A biomolecular force field based on the free enthalpy of hydration and solvation: the GROMOS force-field parameter sets 53A5 and 53A6. J. Comput. Chem. 25, 1656–1676 (2004). 15264259 10.1002/jcc.20090

[b29] Darden T., York D. & Pedersen L. Particle mesh Ewald: An Nlog(N) method for Ewald sums in large systems. J. Chem. Phys 98, 10089–10092 (1993).

[b30] Essmann U. *et al.* A smooth particle mesh Ewald method. J. Chem. Phys. 103, 8577–8593 (1995).

[b31] Jorgensen W. L., Chandrasekhar J., Madura J. D., Impey R. W. & Klein M. L. Comparison of simple potential functions for simulating liquid water. J. Chem. Phys. 79, 926–935 (1983).

[b32] Bussi G., Donadio D. & Parrinello M. Canonical sampling through velocity rescaling. J. Chem. Phys. 126, 014101 (2007). 17212484 10.1063/1.2408420

[b33] Berendsen H. J. C., Postma J. P. M., van Gunsteren W. F., DiNola A. & Haak J. R. Molecular dynamics with coupling to an external bath. J. Chem. Phys. 81, 3684–3690 (1984).

[b34] Hess B., Bekker H., Berendsen H. J. C. & Fraaije J. G. E. M. LINCS: A Linear Constraint Solver for Molecular Simulations. J. Comput. Chem. 18, 1463–1472 (1997).

[b35] Miyamoto S. & Kollman P. A. SETTLE: An analytical version of the SHAKE and RATTLE algorithm for rigid water models. J. Comput. Chem. 13, 952–962 (1992).

[b36] Lemkul J. A. & Bevan D. R. Assessing the stability of Alzheimer’s amyloid protofibrils using molecular dynamics. J. Phys. Chem. B 114, 1652–1660 (2010). 20055378 10.1021/jp9110794

[b37] Kumar S., Bouzida D., Swendsen R. H., Kollman P. A. & Rosenberg J. M. The weighted histogram analysis method for free-energy calculations on biomolecules. I. the method. J. Comput. Chem. 13, 1011–1021 (1992).

[b38] Hub J. S., Groot B. L. d. & Spoel D. v. d. g_whams-A free weighted histogram analysis implementation including robust error and autocorrelation estimates. J. Chem. Theory Comput. 6, 3713–3720 (2010).

[b39] Okamura E., Kakitsubo R. & Nakahara M. NMR determination of the delivery site of bisphenol A in phospholipid bilayer membranes. Langmuir 15, 8332–8335 (1999).

[b40] Tu Y. *et al.* Destructive extraction of phospholipids from Escherichia coli membranes by graphene nanosheets. Nat. Nanotechnol. 8, 594–601 (2013). 23832191 10.1038/nnano.2013.125

