# Supplementary material for: Molecular Dynamics Simulations of the Permeation of Bisphenol A and Pore Formation in a Lipid Membrane
Source: Sci Rep. 2016 Sep 15;6:33399. doi: 10.1038/srep33399 (PMC5024305; doi:10.1038/srep33399)
Supplement: Supplementary Information [file srep33399-s1.doc]

Supplementary Information to

"Molecular Dynamics Simulations of the Permeation of Bisphenol A and Pore Formation in a Lipid Membrane"

by

Liang Chen, Junlang Chen, Guoquan Zhou, Yu Wang, Can Xu & Xiaogang Wang


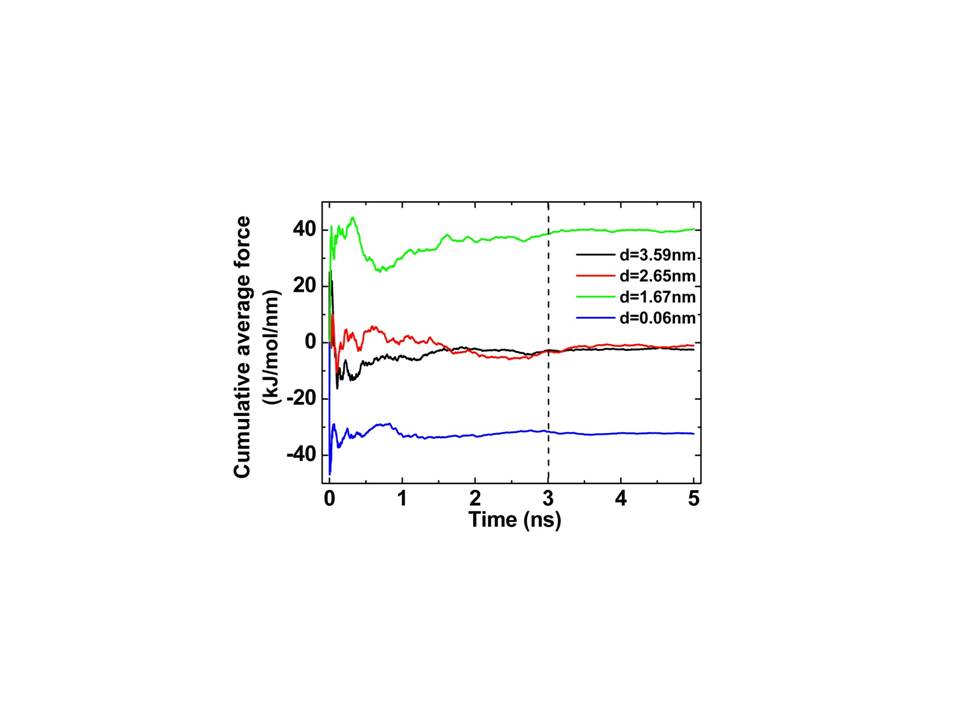


**Figure S1**. Cumulative average forces in the first 5 ns equilibration MD as a function of simulation time at four selected windows. As shown, the fluctuations in the force curves are significant at the beginning, and are dampened after about 3 ns, indicating that the system has reached equilibrium. Therefore, umbrella sampling during the continued 5 ns of production run is well converged.


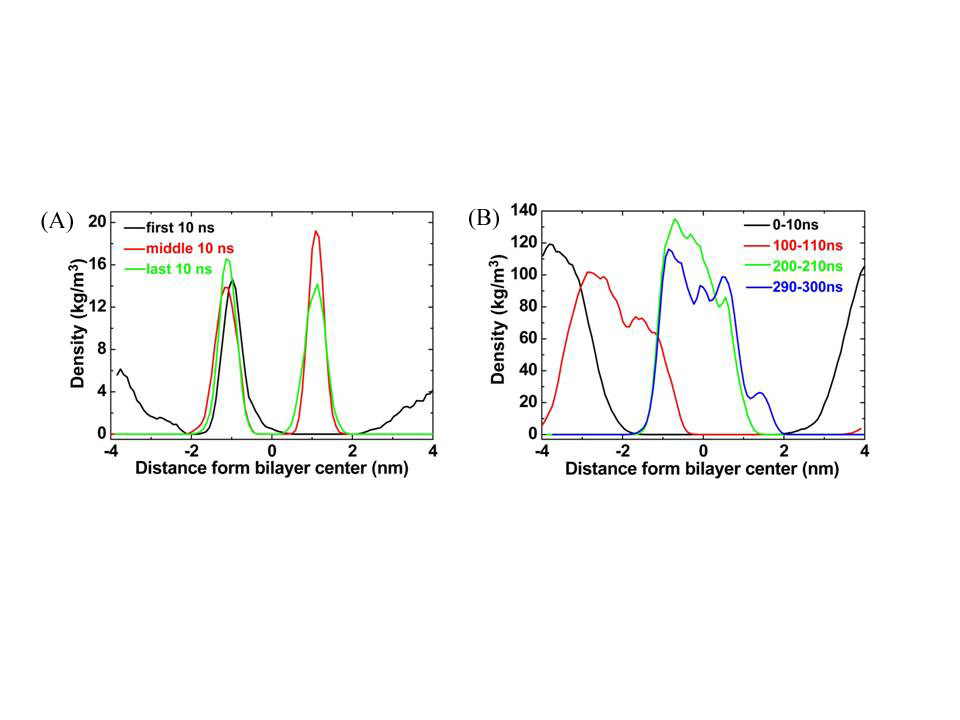


**Figure S2**. Mass density profiles of BPA in the membrane. (A) Two BPA molecules in the symmetric positions. (B) Distribution of BPA cluster in the membrane.

Mass density profile of BPA can be used to evaluate the convergence of the system. As shown in Figure S2A, the peaks of mass density profiles for the middle 10 ns as well as the last 10 ns are in good agreement with each other, implying that the system is well equilibrated at *t*=60 ns. In a similar way, we have compared mass density profile of BPA in the four stages (see Figure R3B). The distribution of BPA in 200-210 ns is close to that in 290-300 ns, though there is a little difference because of thermal disturbance. The system is already equilibrated after 210 ns.


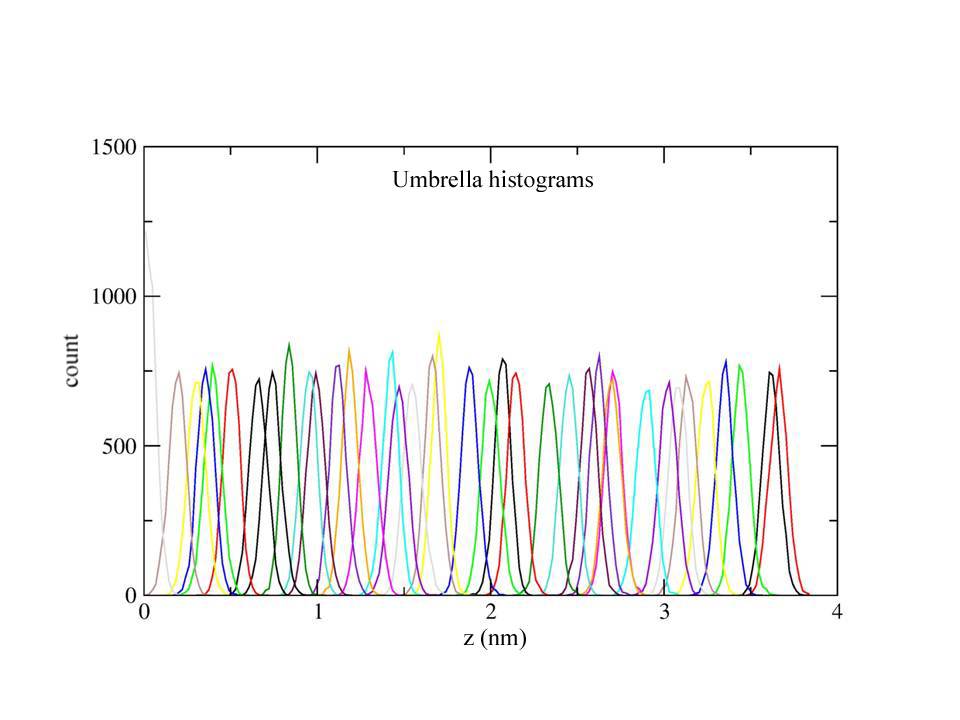


**Figure S3.** Umbrella histograms. As shown, the overlap between windows from about 0-3.5 nm of COM spacing is reasonable, and the umbrella sampling is sufficient.


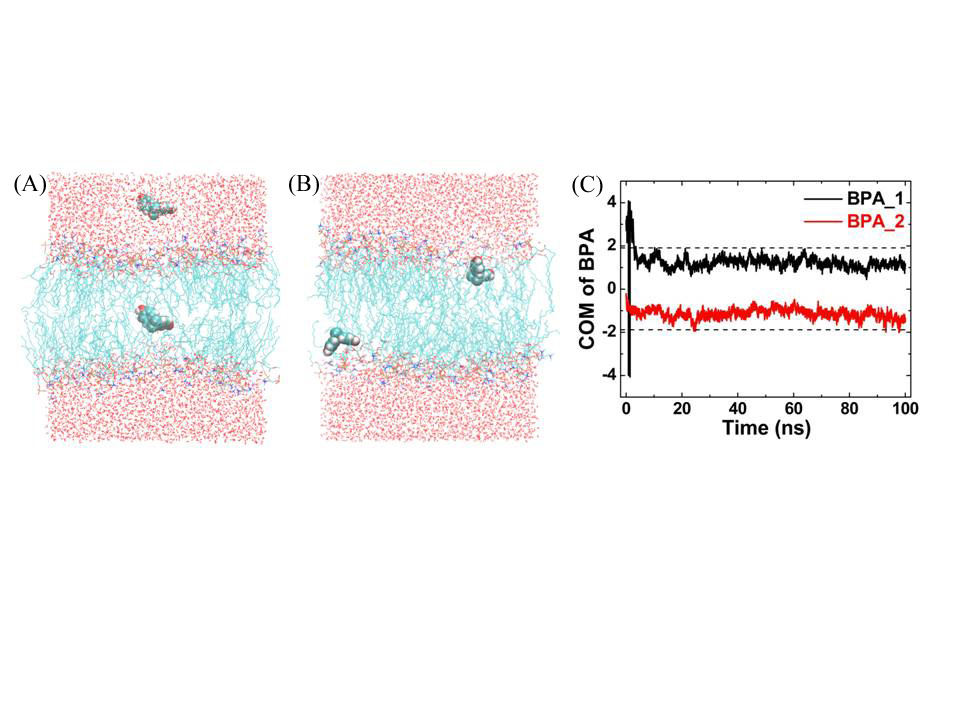


**Figure S4.** A representative trajectory of the translocation of BPA across a lipid bilayer with new partial charges, obtained by DFT calculations at wb97xd/6-31+g(d, p) level of theory. (A) The initial configuration. (B) The final snapshot. (C) The z-coordinates of the COMs of BPA vs. simulation time. The results are in good agreements with those in the paper.
